# Supplementary material for: Identifying technologies in circular economy paradigm through text mining on scientific literature
Source: PLoS One. 2024 Dec 27;19(12):e0312709. doi: 10.1371/journal.pone.0312709 (PMC11676554; doi:10.1371/journal.pone.0312709)
Supplement: Attachment — Submitted filename: Response to Reviewers.docx [file pone.0312709.s004.docx]

To address the reviewers' comments, we have separated the text of the review into individual comments, each highlighted in “black”. For each comment, we provide a response highlighted in “**blue**”, using **bold** text for clarity, and where necessary, we include parts of the paper's text in “*blue*” *italic*. Additionally, in the manuscript, we have tracked each change in “red” color to clearly indicate the modifications made in response to the reviewers' feedback.

# Reviewer #1

## R1.1 Comment

**Related work part has been appended at the end of the introduction section, but it is not providing a detailed review of state of the art. The authors should make a separate section/subsection on related works and present similar works. A table should be also provided to summarize the outcomes of these studies.**

**We apologize for any confusion regarding the placement of the "Related Work" section. In our original manuscript, the related works were indeed presented in a separate section, not appended at the end of the introduction. However, we realize that this might not have been clear due to the lack of a detailed description of the paper's structure. Additionally, the section title may have contributed to this misunderstanding.**

**To improve clarity, we have now added a description of the paper's structure in the introduction, which outlines the organization of the manuscript, including the placement of the "Related Work" section. Furthermore, we have retitled this section to better reflect its content and purpose. We added the organization of the paper as follows:**

*“The rest of the paper is structured as follows. Section 2 provides a description of the related works on CE and discusses the main NLP methods used to map a technological landscape. In Section 3, we explain the method we used. Section 4 shows the main insights and results of the paper. Section 5 proposes some implications that emerge from our work for researchers, practitioners and policy makers and details the main limitations of the proposed approach.”*

**In response to your suggestion related to the table, we have also included a table summarizing the outcomes of these studies, which should provide a concise overview of the existing literature and our work's positioning within it. The table is Table 1.**

**We are grateful for your insights, which have helped us to improve the clarity and comprehensiveness of our manuscript. We believe these revisions address your concerns and enhance the overall quality of our paper.**

## R1.2 Comment

**The key part of the introduction section is missing; the author should include the Contribution section. In the last paragraph, the authors must mention the organization of the paper.**

**We have addressed the key points you raised by incorporating a contribution paragraph (as also suggested by the second reviewer, see Comment 2.1) into the introduction and detailing the organization of the paper in the last paragraph of the introduction. Below are the main contributions of our paper and an outline of its organization:**

*“The identification of these technologies will significantly contribute to advancing the CE agenda in several ways. By comprehensively analyzing a broad array of technological domains within the CE, our research will uncover areas that have been underexplored or neglected. This identification of gaps in current research and technological implementation provides a clear roadmap for future investigations, ensuring that all technological aspects of CE are addressed and optimized.*

*Highlighting the technological advancements and their applications within the CE framework will inspire innovation across various sectors. Understanding how different technologies can be integrated and utilized in CE practices will encourage researchers and businesses to develop new solutions and improve existing processes. This innovation is crucial for achieving the efficiency and sustainability goals of the CE. Furthermore, the study can facilitate collaboration among different stakeholders. Researchers, businesses, and policymakers can use this information to work together on integrated solutions that span multiple sectors and technological domains.*

*A comprehensive understanding of the technological landscape in the CE will provide policymakers with the necessary insights to create informed and effective regulations and incentives. By identifying which technologies are most promising and which areas need more support, policymakers can tailor their strategies to promote sustainable practices, drive technological adoption, and ensure coherent and supportive regulatory frameworks. Moreover, the comprehensive analysis of CE technologies can support funding and investment decisions by providing evidence of the viability and impact of these technologies. Investors and funding agencies can use this information to allocate resources more effectively towards promising CE innovations.*

*The rest of the paper is structured as follows. Section 2 provides a description of the related works on CE and discusses the main NLP methods used to map a technological landscape. In Section 3, we explain the method we used. Section 4 shows the main insights and results of the paper. Section 5 proposes some implications that emerge from our work for researchers, practitioners and policy makers and details the main limitations of the proposed approach.”*

## R1.3 Comment

**The novelty of this paper is not clear. The difference between present work and previous Works should be highlighted.**

**Thank you for your valuable feedback. We acknowledge that the novelty of our paper was not clearly articulated in the previous version of the manuscript. In response to your comment, we have added a dedicated paragraph explicitly highlighting the novel contributions of our work. Below is an outline of this newly added content:**

*“The novelty of our paper lies in several key areas. First, unlike many existing papers that predominantly concentrate on individual technologies or industry domain in the CE topic, our paper presents a holistic view of the CE, addressing a broader spectrum of technological contexts.*

*Second, while a significant portion of the literature emphasizes the examination of digital technologies within the CE, leaving other technological domains relatively unexplored, our research expands beyond this narrow focus. By exploring a wide array of technological domains, we address the gaps left by existing literature and offer a more inclusive examination of CE technologies.*

*Third, our paper employs innovative research methods that move beyond traditional literature reviews, which often rely on expert opinions and can introduce biases. We utilize advanced automated techniques to enhance objectivity and depth in our analysis, thus mitigating potential biases.*

*Finally, even when scientific articles use automated techniques like NLP, their focus tends to be narrow and does not analyze the CE challenge comprehensively from a technological perspective. In contrast, our paper employs these automated techniques to provide a comprehensive technological analysis, addressing the broader implications and interactions within the CE landscape.”*

## R1.4 Comment

**The figures in the article have not been appropriately described and discussed. A brief discussion of each figure should be provided for better visibility and clarity of findings**

**We have added a detailed description and discussion for each figure to enhance visibility and clarity of our findings. These additions provide a comprehensive understanding of the visual data, ensuring that the insights and implications of our analysis are clearly communicated.**

**Specifically, we added the following text in Section 4.1:**

*“Table 3 presents detailed information about the top-15 fastest-growing technologies identified using our NLP method. For each technology, the table includes several key metrics. The total number of papers indicates how many scientific papers have mentioned or discussed the technology in the context of the CE. The percentage of papers shows the proportion of papers on this specific technology relative to the total number of papers we retrieved. Additionally, the table provides the number of papers published in two distinct periods: from 2015 to 2020 and from 2021 to 2023. This division allows us to see the historical and recent interest in each technology. The RDGR measures the growth rate of the technology. It is calculated by dividing the number of papers published in the last three years (2021-2023) by the number of papers published in the preceding six years (2015-2020), as explained in Section 3.4. A higher RDGR indicates a faster-growing technology.*

*To better understand how to read Table 3, consider the example of the technology "Digital Twins." This technology is mentioned in 19 scientific papers, which constitutes 0.04% of all papers focused on CE. Between 2015 and 2020, only one paper discussed digital twins. However, between 2021 and 2023, this number increased to 18 papers. The RDGR for digital twins is 18.00. This means that in recent years (2021-2023), the number of papers discussing digital twins is 18 times higher compared to the previous years (2015-2020). The example demonstrates that initially, digital twins were scarcely mentioned in the literature (only one paper from 2015 to 2020). However, its prominence has sharply increased recently, with 18 papers published from 2021 to 2023. The high RDGR underscores its rapid emergence and growing importance.*

*This table provides a comprehensive overview of the growth trends of these technologies over time. By including these specific metrics, it allows for easy comparison between different technologies, highlighting which ones are emerging more rapidly in the field of the circular economy.”*

**and this in the Section 4.2:**

*“Figure 1 provides a visual representation of the clustering of technologies related to the CE. Each node in the graph represents a specific technology identified in our analysis. The size of a node indicates the centrality or importance of the technology within the network. Centrality in this context is calculated using a specific metric known as degree centrality. The degree centrality measures the number of direct connections a node (technology) has to other nodes in the network. In other words, it counts how many times a specific technology co-occurs with other technologies in the scientific papers analyzed. Larger nodes represent technologies that are more central and interconnected with other technologies. The lines connecting the nodes are known as edges. They represent the co-occurrence of technologies within the same scientific papers. The thickness of an edge indicates the strength of the connection between two technologies. Thicker edges mean a higher frequency of co-occurrence. The nodes are grouped into clusters, which are color-coded. Each cluster represents a distinct technological area within the CE field. The clustering algorithm used (Louvain method as described in Section 3.4) identifies these groups based on the strength of connections within the network, ensuring that technologies frequently mentioned together are grouped into the same cluster.*

*Figure 1 and related Table 4 show the results of network analysis performed on technologies extracted from scientific papers. Each cluster is shown in Figure 1 using different colors.*

*The content of each cluster is shown in Table 4, where a comprehensive overview of the technologies within each cluster is presented. Each cluster is given a label based on the most common and relevant terms within the cluster. These labels are derived from the prominent tokens or terms occurring in each cluster but are manually assigned by the authors to ensure they accurately reflect the thematic focus of the technologies grouped together. This careful labelling helps readers quickly understand the key technological themes represented in each cluster. For each cluster we can see the label of the cluster, the number of technologies within the cluster (Size), the number of technologies that are considered obsolescent (No. of Obsolescent Techs), the number of technologies that are considered emergent (No. of Emergent Techs), the mean RDGR of the technologies (Mean GI), the standard deviation of the RDGR (SD), the top 15 technologies based on degree centrality measure, so technologies that have the highest number of connections to other technologies in the network, making them the most central and influential within their cluster.*

*To determine if a technology is obsolescent, we look at its RDGR. Technologies with an RDGR lower than 1 and close to 0 are considered obsolescent. This low RDGR indicates that the number of papers discussing these technologies has decreased significantly in recent years compared to previous years, suggesting a decline in their relevance or usage. Conversely, if the RDGR is higher than 1 and tends to be very high, the technology is considered emerging. A high RDGR indicates a significant increase in the number of papers discussing the technology in recent years compared to previous years, signifying a growing interest and relevance in the field. For example, technologies like "Digital Twins" with an RDGR of 18.00 demonstrate rapid growth, highlighting their emerging importance within the circular economy.*

*Similarly, the mean of RDGR related to each technological cluster provides an average growth rate for the technologies, indicating how rapidly the technologies are gaining attention. This measure gives us an overview of whether the technological cluster is obsolescent or emerging and how quickly the cluster is growing on average.*

*By analyzing Figure 1 and Table 4 together, you can gain a detailed understanding of how various technologies are interconnected and how they are grouped into thematic clusters. This combined analysis highlights the central technologies driving the CE and identifies both emerging and declining trends within the field.*

*The results of the network analysis are presented in a visual format and can be accessed through the following link: https://vitogiordano94.github.io/Technologies-in-Circular-Economy/network_analysis/. This link will take you to a webpage where you can view the network and interact with it. In the link, the reader can zoom in and out using his/her mouse scroll wheel or the webpage’s zoom controls and pan across the network by clicking and dragging the background. The reader can also search a specific node (technology) in the searching box to see detailed information about the technology it represents, and click on a node to fix its position and highlight its connections.”*

## R1.5 Comment

**The conclusion section needs to elaborate more by discussing the disadvantages of the developed model & discussion on the results obtained. The author should also include the future work section.**

**In response to your comment, we have further elaborated on the conclusion of our work. We added a paragraph on the discussion of the obtained results:**

*“In this study, we have utilized advanced NLP techniques to investigate the technological landscape within the CE paradigm. Our analysis of over 45,000 scientific publications has provided significant insights into the emerging and prevailing technologies driving the transition towards a more sustainable and circular economy.*

*The findings revealed a strong emphasis on digital technologies, life cycle assessment methodologies, and biomaterials. Through the identification of seven distinct technological domains, we were able to categorize the various technological advancements and their applications within the CE field. These domains include Digital Technologies, New Processes and Materials, and Assessment Technologies, each playing a crucial role in enhancing resource efficiency, reducing waste, and promoting sustainable industrial practices. Our network analysis, visually represented in Figure 1 and detailed in Table 4, illustrates the interconnectedness of these technologies and their relative importance within the CE landscape. Technologies such as Digital Twins, Machine Learning, and Internet of Things (IoT) were identified as highly central and rapidly emerging, signifying their increasing relevance and application across various sectors. On the other hand, technologies with lower RDGR were considered obsolescent, indicating a decline in their significance.*

*One of the key contributions of our research is the development of RQ3, which specifically addresses the industrial implications of these technological changes. By examining how these technologies can be practically implemented and integrated into existing systems, we have provided valuable insights into their potential to promote the circular economy. In Section 4.3, we highlight specific insights into the advantages and challenges of adopting these technologies at an industrial level. For instance, Digital Twins, identified as an emerging technology with a high RDGR, offer significant benefits such as improved efficiency, enhanced decision-making, cost savings, and sustainability. However, they also pose challenges including data accuracy and integration, data security, and organizational changes. Similarly, Virtual Reality technology provides immersive experiences and resource-saving opportunities but faces issues related to motion sickness, cost, and accessibility. These detailed insights into each technology's advantages, challenges, and fields of application underscore the practical implications and guide industries in effectively integrating these technologies to advance the circular economy.”*

**added a text about the limitations of our work:**

*“The paper is subject to several limitations, including the scope of observation, methodological challenges, data quality and bias issues, and limitations of text mining algorithms. Firstly, the scope mainly focuses on scientific articles that emphasize technological aspects. However, it is important to note that a significant source of information on technologies resides within patents, while scientific publications only give us the technical perspective of researchers. Identifying relevant patents in the CE domain can be challenging, and crafting effective search strategies for CE-related patents remains a complex task. Some attempts have been made to capture patents related to CE, for instance the “green patent database” , but it covers only a fraction of CE aspects, related to environmental sustainability. Furthermore, patents tend to underrepresent the economic and social sustainability facets of CE. For these reasons, one potential future development is the identification of relevant patents set for mapping the technological landscape of CE.*

*Secondly, the methodology adopted for technology extraction, explained in Puccetti et al. (2023), presents certain precision and recall challenges. Precision concerns whether the terms we extracted effectively represent technologies. Recall pertains to whether we might have missed certain technologies. These challenges carry an element of uncertainty, since the method was originally designed for patents, not scientific articles. We have attempted to address precision issues by manually reviewing the results. To mitigate recall problems, we have sought to enhance our analysis through collaboration with domain experts, including two of the authors of this paper. Nevertheless, there is a requirement for increased efforts to effectively tackle these challenges.*

*Thirdly, data quality issues also pose a limitation, as the accuracy and comprehensiveness of datasets used for NLP can impact the results. In the current paper, we use a query-based approach, but the precision and recall of the query in identifying all and only the relevant scientific articles on CE is not fully explored. "Identifying all relevant articles" means that the query should retrieve every pertinent paper within the field, without missing any significant studies. Conversely, "identifying only relevant articles" means that the query should exclude any unrelated papers, ensuring that all retrieved articles are pertinent to the CE analysis. This can be problematic for the analysis because of the principle "garbage in, garbage out". Future efforts should focus on improving data quality through rigorous cleaning and validation procedures.*

*Finally, the limitations of NLP algorithms, such as misinterpretation of context and ambiguity in language, can also affect the results. This ambiguity issue is more pronounced in technical language, such as in scientific articles or patent documents and descriptions of technology (Puccetti et al., 2023; Giordano et al., 2021), since whether a word or phrase pertains to technology heavily depends on the context in which it appears. Currently, our algorithm does not manage this aspect effectively. However, in the future, advanced NLP algorithms that rely on deep learning methods and are capable of considering context (such as BERT and other contextual word embeddings algorithms, as well as Large Language Models) are needed to fully analyse and interpret the data accurately.”*

**and added a section on the main future directions:**

*“Next steps include conducting a comprehensive analysis of patents related to CE technologies to capture innovations not extensively covered in scientific literature. Expanding the scope of data sources to include industry reports, grey literature, and real-world case studies can help mitigate biases and provide a more balanced perspective. Implementing rigorous data cleaning and validation procedures will enhance accuracy and reliability. Exploring advanced natural language processing techniques and machine learning models will improve precision and recall in technology extraction. Finally, engaging with experts from various fields will help refine methodologies and ensure a comprehensive understanding of CE technologies that can lead in translating research findings into practical recommendations for policymakers and industry practitioners.”*

# Reviewer #2

## R2.1 Comment

**Highlighting the potential contribution of the study is essential. How will the identification of these technologies contribute to advancing the circular economy agenda? Will it help in identifying gaps, fostering innovation, or guiding policy decisions?**

**We have addressed this important issue you raised by incorporating a contribution paragraph (as also suggested by the first reviewer, see Comment 1.2) into the introduction:**

*“The identification of these technologies will significantly contribute to advancing the CE agenda in several ways. By comprehensively analyzing a broad array of technological domains within the CE, our research will uncover areas that have been underexplored or neglected. This identification of gaps in current research and technological implementation provides a clear roadmap for future investigations, ensuring that all technological aspects of CE are addressed and optimized.*

*Highlighting the technological advancements and their applications within the CE framework will inspire innovation across various sectors. Understanding how different technologies can be integrated and utilized in CE practices will encourage researchers and businesses to develop new solutions and improve existing processes. This innovation is crucial for achieving the efficiency and sustainability goals of the CE. Furthermore, the study can facilitate collaboration among different stakeholders. Researchers, businesses, and policymakers can use this information to work together on integrated solutions that span multiple sectors and technological domains.*

*A comprehensive understanding of the technological landscape in the CE will provide policymakers with the necessary insights to create informed and effective regulations and incentives. By identifying which technologies are most promising and which areas need more support, policymakers can tailor their strategies to promote sustainable practices, drive technological adoption, and ensure coherent and supportive regulatory frameworks. Moreover, the comprehensive analysis of CE technologies can support funding and investment decisions by providing evidence of the viability and impact of these technologies. Investors and funding agencies can use this information to allocate resources more effectively towards promising CE innovations.”*

## R2.2 Comment

**Discussing the practical implications of the findings is crucial. How can the identified technologies be practically implemented in industries or integrated into existing systems to promote the circular economy?**

**We agree that discussing the practical implications of our findings is crucial. In the previous version of our manuscript, the key practical implications of our research might not have been expressed clearly enough. However, we want to highlight that our research question RQ3 – "How does this technological change impact at the industrial level?" – was specifically developed to address and ensure the industrial implications of our work. In fact, Section 4.3 (that answer to the RQ3) includes a strong focus on how the identified technologies can be practically implemented in industries and integrated into existing systems to promote the circular economy. In the current version of the manuscript, we emphasize this aspect more clearly and highlight that RQ3 provides key practical implications.**

**For this reason we included in the Introduction (Section 1) the following text:**

*“The findings of our analysis reveal a strong emphasis on emerging digital technologies. Additionally, we observe a growing research focus on technologies associated with lifecycle assessment and biomaterials. Furthermore, we have identified seven distinct technological domains within the CE field. Finally, we provide the key advantages and challenges pertaining to the adoption of these technologies within the CE framework and explain the main fields of potential application. In fact, RQ3 is designed to provide key practical implications by identifying the main issues and benefits associated with adopting these technologies. This focus ensures that our findings are not only theoretically significant but also practically applicable, guiding industries in integrating these technologies to promote a circular economy effectively.”*

**and in the Section 4.3:**

*“Answering RQ3 helps us understand the practical implications of the identified technologies, focusing on the main issues and benefits of their adoption. This section delves into how these technologies can be practically implemented in industries or integrated into existing systems to promote the circular economy. By examining the real-world impact of these technological changes, we aim to provide clear guidance for industries looking to adopt these innovations.”*

## R2.3 Comment

**Acknowledging the challenges and limitations of text mining and the study itself is important. For instance, biases in the literature, data quality issues, or limitations of text mining algorithms should be addressed.**

**Thank you for your valuable feedback. In response to your comment, we have further elaborated on the limitations of our work. Below is text we added:**

*“The paper is subject to several limitations, including the scope of observation, methodological challenges, data quality and bias issues, and limitations of text mining algorithms. Firstly, the scope mainly focuses on scientific articles that emphasize technological aspects. However, it is important to note that a significant source of information on technologies resides within patents, while scientific publications only give us the technical perspective of researchers. Identifying relevant patents in the CE domain can be challenging, and crafting effective search strategies for CE-related patents remains a complex task. Some attempts have been made to capture patents related to CE, for instance the “green patent database” , but it covers only a fraction of CE aspects, related to environmental sustainability. Furthermore, patents tend to underrepresent the economic and social sustainability facets of CE. For these reasons, one potential future development is the identification of relevant patents set for mapping the technological landscape of CE.*

*Secondly, the methodology adopted for technology extraction, explained in Puccetti et al. (2023), presents certain precision and recall challenges. Precision concerns whether the terms we extracted effectively represent technologies. Recall pertains to whether we might have missed certain technologies. These challenges carry an element of uncertainty, since the method was originally designed for patents, not scientific articles. We have attempted to address precision issues by manually reviewing the results. To mitigate recall problems, we have sought to enhance our analysis through collaboration with domain experts, including two of the authors of this paper. Nevertheless, there is a requirement for increased efforts to effectively tackle these challenges.*

*Thirdly, data quality issues also pose a limitation, as the accuracy and comprehensiveness of datasets used for NLP can impact the results. In the current paper, we use a query-based approach, but the precision and recall of the query in identifying all and only the relevant scientific articles on CE is not fully explored. "Identifying all relevant articles" means that the query should retrieve every pertinent paper within the field, without missing any significant studies. Conversely, "identifying only relevant articles" means that the query should exclude any unrelated papers, ensuring that all retrieved articles are pertinent to the CE analysis. This can be problematic for the analysis because of the principle "garbage in, garbage out". Future efforts should focus on improving data quality through rigorous cleaning and validation procedures.*

*Finally, the limitations of NLP algorithms, such as misinterpretation of context and ambiguity in language, can also affect the results. This ambiguity issue is more pronounced in technical language, such as in scientific articles or patent documents and descriptions of technology (Puccetti et al., 2023; Giordano et al., 2021), since whether a word or phrase pertains to technology heavily depends on the context in which it appears. Currently, our algorithm does not manage this aspect effectively. However, in the future, advanced NLP algorithms that rely on deep learning methods and are capable of considering context (such as BERT and other contextual word embeddings algorithms, as well as Large Language Models) are needed to fully analyse and interpret the data accurately.”*
